# A New Method for Spatial Estimation of Water Quality Using an Optimal Virtual Sensor Network and In Situ Observations: A Case Study of Chemical Oxygen Demand

**DOI:** 10.3390/s23104739

**Published:** 2023-05-14

**Authors:** Na Zhao

**Affiliations:** 1 State Key Laboratory of Resources and Environmental Information System, Institute of Geographic Sciences and Natural Resources Research, Chinese Academy of Sciences, Beijing 100101, China; zhaon@lreis.ac.cn; 2 College of Resources and Environment, University of Chinese Academy of Sciences, Beijing 100101, China; 3 Jiangsu Center for Collaborative Innovation in Geographic Information Resource Development and Application, Nanjing 210023, China

**Keywords:** water quality, estimation, accuracy, Poyang Lake

## Abstract

Accurate water quality estimation is important for water environment monitoring and water resource management and has emerged as a pivotal aspect of ecological rehabilitation and sustainable development. However, due to the strong spatial heterogeneity of water quality parameters, it is still challenging to obtain highly accurate spatial patterns of them. Taking chemical oxygen demand as an example, this study proposes a novel estimation method for generating highly accurate chemical oxygen demand fields in Poyang Lake. Specifically, based on the different water levels and monitoring sites in Poyang Lake, an optimal virtual sensor network was first established. A Taylor expansion-based method with integration of spatial correlation and spatial heterogeneity was developed by considering environmental factors, the optimal virtual sensor network, and existing monitoring stations. The proposed approach was evaluated and compared with other approaches using a leave-one cross-validation process. Results show that the proposed method exhibits good performance in estimating chemical oxygen demand fields in Poyang Lake, with mean absolute error improved by 8% and 33%, respectively, on average, when compared with classical interpolators and remote sensing methods. In addition, the applications of virtual sensors improve the performance of the proposed method, with mean absolute error and root mean squared error values reduced by 20% to 60% over 12 months. The proposed method provides an effective tool for estimating highly accurate spatial fields of chemical oxygen demand concentrations and could be applied to other water quality parameters.

## 1. Introduction

Freshwater plays a critical role in humans, and the global freshwater supply available for human utilization and consumption is severely limited, accounting for only 2% of the Earth’s total water content, despite the fact that water covers approximately 70% of the planet’s surface [1,2]. However, the quality of freshwater in numerous inland lakes is under severe threat and degradation as a result of various anthropogenic factors, such as land-use changes, discharge of untreated sewage, non-point source pollution from urban and agricultural regions, and other human activities [3,4,5,6]. In recent decades, a predominant trend observed in lakes worldwide is the escalation of organic matter concentrations [7]. Elevated levels of organic matter in surface water can have deleterious effects on the structure and functioning of aquatic ecosystems, posing a significant threat to the stability of lake ecosystems and limiting the sustainable development of urban areas adjacent to lakes. Accurate assessment of the spatial distribution of water quality is a crucial prerequisite for understanding and mitigating environmental risks associated with organic matter accumulation in freshwater systems. Therefore, developing reliable and efficient methods for estimating and monitoring spatial patterns of water quality is a fundamental task that requires interdisciplinary efforts by researchers, policymakers, and stakeholders. In addition, in the context of increasingly stringent pollution control and water quality improvement policies, the precise estimation of water quality is of paramount importance in assessing the effectiveness of water resource management strategies, allowing decision-makers to anticipate the response of lake ecosystems to different management scenarios [8].

Chemical oxygen demand (COD) is a crucial parameter that provides valuable information on the condition of discharged pollutants and the level of organic pollution present in aquatic environments [9]. Acquiring the spatial distribution of COD is essential for gaining a more profound comprehension of the biogeochemical mechanisms underlying organic pollutant matter in aquatic ecosystems [10]. However, the intricate composition of organic matter and the complexity of its transformation mechanisms result in a highly heterogeneous spatiotemporal pattern of COD dynamics in water bodies. This variability poses significant challenges in accurately estimating COD levels [11]. Traditionally, the primary approach to monitoring COD in aquatic ecosystems has been through in situ sample collection and laboratory measurements. However, this method is time-consuming, and the resulting data have low temporal and spatial resolutions, thus providing only discrete data points [12]. More importantly, conventional ground monitoring methods are inadequate in capturing water quality parameters with high spatial–temporal resolution across lakes. Furthermore, monitoring water quality is becoming increasingly challenging due to the resource-intensive nature of sampling tasks and the sheer number of chemicals that are discharged into inland waters from various industrial and domestic sources. Presently, the estimation of COD primarily relies on chemical methods [12,13], which can yield accurate results, but result in secondary pollution. The interpolation method is a widely adopted technique for generating spatial COD fields from limited sample sites. However, the accuracy of COD estimates based on interpolation is heavily reliant on the station network density and the degree of spatial heterogeneity of COD. In cases where the station network is sparse and the spatial heterogeneity of COD is high, it can be challenging to obtain accurate spatial information of COD using interpolation-based methods. Another basic approach required to solve the water pollution problem is the modeling of water quality changes by developing some mathematical models [14,15]. These process-based models have the ability of simulating and predicting complex processes in water ecosystems, identifying the behavior of pollutants, and recognizing the spatial distribution of water quality parameters [16,17]. However, due to the different theories and algorithms used in the models, the modeling outputs of different models have big differences. In addition, water quality modeling is challenging due to insufficient representative site selection and sample gaps, lack of calibration, errors in data reporting, and parameterization [17,18,19].

In recent decades, remote sensing technology has provided a promising way for lake water quality continuous monitoring at local scales, which is an ideal method for monitoring aquatic environments because it allows interpretation of received radiance at multiple wavelengths, thereby enabling long-term monitoring of water quality parameters [11,20,21]. Numerous studies have focused on applying remote sensing techniques to obtain water quality parameters. However, most of these studies have primarily concentrated on optically active parameters, such as Chlorophyll-a, dissolved colored organic matter, and turbidity. In contrast, less attention has been paid to non-optically active parameters, which are less likely to influence the optical properties measured using remote sensing [22,23,24]. As non-optical water quality parameters, the estimation of COD through remote sensing is challenging due to the fact that changes in COD levels may not result in observable changes in water color, making it difficult to capture directly from satellite observations. As a result, retrieving accurate and reliable COD data using remote sensing remains a significant challenge. Considerable researches have been concentrated on the estimation of COD using remote sensing methods, of which the indirect method is the most accepted and applied one, which is established based on the observation of a strong correlation between optically and non-optically active parameters [20,24,25]. At present, the use of machine learning regression methods for remote sensing retrieval of COD has been increasingly adopted due to their ability to address complex nonlinear problems in estimating non-optically active water quality parameters [12,23,26,27]. However, the performance of machine learning methods greatly depended on the training data and the robustness of them varies largely among different regions and datasets [28]. In addition, the application of satellite remote sensing in local water environments is limited by several factors, such as coarse spatial resolution, weather conditions, and low signal-to-noise ratios [29,30,31,32].

Poyang Lake (PL) is the largest freshwater lake in China, and wetlands in PL basin are one of the typical global river-lake silted freshwater wetlands, playing an important role in preserving ecological function. It has been reported that the self-purification capacity of PL has been weakened in recent years and water quality is deteriorating due to extensive human activities [33]. However, the literature on estimating water quality parameters in PL is still limited, and no studies have attempted to focus on the simulation of COD in PL. This paper aims to propose a new estimation method for generating spatial distribution of COD in PL. First, considering the sparse site observations, some virtual sensors are established by combining Shannon’s entropy and semi-variogram using the available monitoring stations and local topographical characteristics. The values of the virtual sensors are retrieved using RF method combined with the recently launched Sentinel-2 imagery. Second, taking into account the spatial correlation and spatial heterogeneity, the estimation of COD was obtained by proposing a novel kernel regression method based on Taylor expansion, using explanatory variables, the values from virtual sensors and monitoring stations. This study provides a more efficient water quality spatial estimation approach, which will facilitate water resource management and policy making. The subsequent sections of this research paper are structured as follows. Section 2 gives a comprehensive account of the materials employed in the study, including information on the study area, datasets used for the analysis, and the proposed method. The results are shown in Section 3. Discussions and conclusions are given in Section 4 and Section 5, respectively.

## 2. Materials and Methods

### 2.1. Study Area and Data

**Figure 1:**
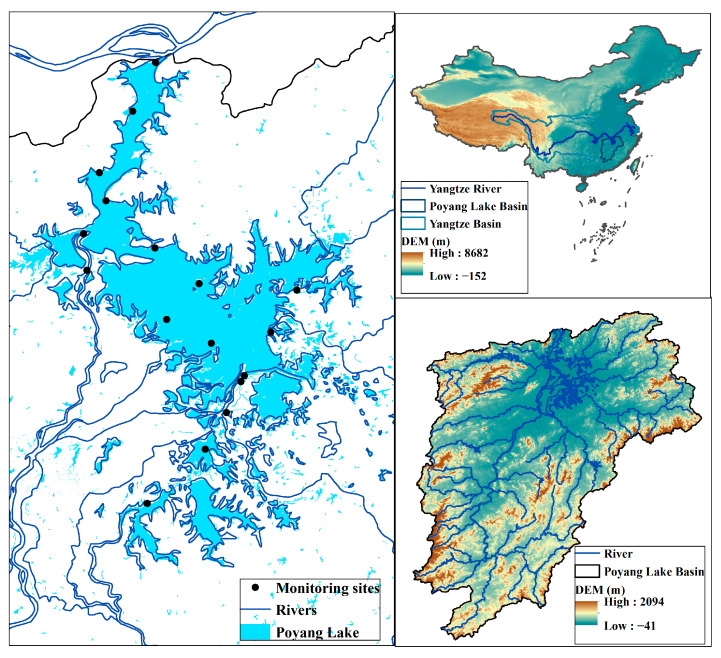
Locations of COD measurements and the Poyang Lake boundary.

Located at 28°22′ N~29°25′ N and 115°47′ E~116°45′ E, PL holds the distinction of being the most significant freshwater lake in China. It features a long and slender river in the northern region that connects it to the Yangtze River, with the southern region being the primary zone. The elevation of the PL exhibits a gradual increase pattern from the north to south and west to east, as shown in Figure 1. Since the lake is a tributary of the Yangtze River, there is a direct exchange and interaction between the two water bodies [34]. The primary factors governing the water level of PL are the Yangtze River and the “five rivers”, namely Gan River, Fu River, Xin River, Rao River, and Xiu River. The water levels of these rivers have a considerable impact, with fluctuations of over 10 m observed in some cases [35]. As a seasonal lake, the water level displays a distinct pattern wherein it rises primarily during the months of April to June, owing to the influx of water from the five rivers. Subsequently, from July to October, the backflow from the Yangtze River also contributes to a rise in water levels. Following this, the water levels gradually receded, starting in October and continuing for approximately six months [36]. The topographical features of PL and changes in the flow of the five rivers significantly affect the lake’s surface area and water level, leading to substantial variations across seasons. The lake experiences seasonal fluctuations in its water volume, with levels varying between the winter and summer periods. Notably, the water depth increased to 19.4 m in August but declined to 7.9 m in January. PL is a crucial source of drinking water, irrigation water, aquaculture water, and industrial water. Additionally, it plays a pivotal role in regulating river water levels, preserving water resources and maintaining the ecological equilibrium of the neighboring regions. The lake’s water quality is of the utmost significance, particularly as a source of drinking water for human consumption. However, the water quality of PL is affected by human activities such as dredging, transportation, and agriculture. In recent decades, the lake’s self-purification capacity has weakened, leading to a decline in water quality [37].

A total of 14 water samples were collected from PL in the year 2021 (Figure 1). The data collected from these samples were incorporated into the analysis process to enhance the accuracy of the estimations. The water quality parameters were measured by monitoring centers and subjected to quality control before being promptly transmitted to the server via GPRS. The COD values of all samples were determined using conventional chemical methods in the laboratory and were considered the actual values for analysis.

**Figure 2:**
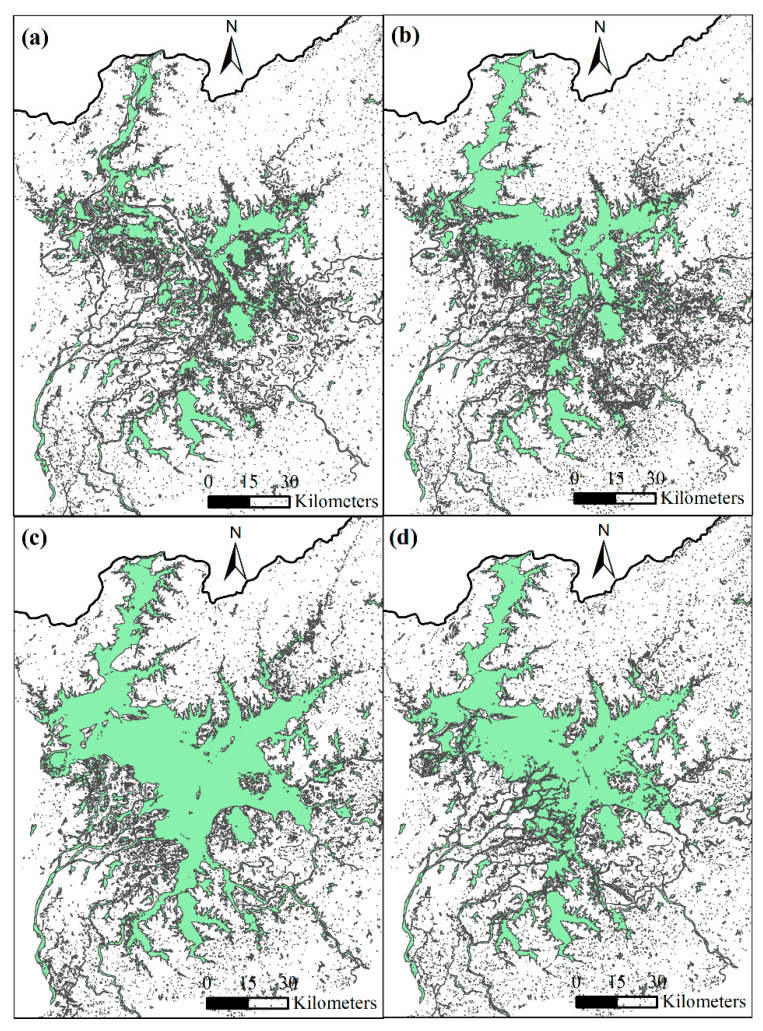
The water area of PL in (**a**) January, (**b**) April, (**c**) July, and (**d**) October, 2021.

The European Space Agency (ESA) offers real-time updated Sentinel-2 MultiSpectral Instrument (MSI) imagery, which can be freely downloaded from https://scihub.copernicus.eu (accessed on 18 January 2023). This imagery is composed of thirteen spectral bands, spanning from the visible (VNIR) and near-infrared (NIR) to short-wave infrared (SWIR). The spatial resolution of these bands varies from 10 m to 60 m. For this research, atmospheric apparent reflectance products were used after ortho-rectification and sub-pixel geometric correction. The water area over different months (as shown in Figure 2) was determined by extracting the normalized difference water index (NDWI, [38]):

(1) $$\mathrm{NDWI}=\frac{X_{Green}-X_{NIR}}{X_{Green}+X_{NIR}}$$

where *X_NIR_* and *X_Green_* are the grid values of the *N_IR_* band and the green band, respectively. For Sentinel-2 imagery, the NIR band and green band are B8 and B3, respectively.

### 2.2. Meteorology

**Figure 3:**
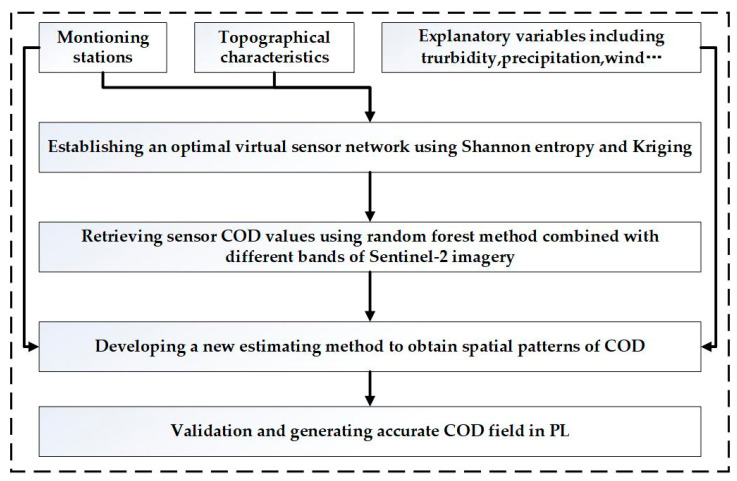
The flowchart of the proposed approach.

In this research, we first use the Shannon’s entropy and semi-variogram function to establish an optimal virtual sensor network, and use random forest (RF) method, combing with the Sentinel-2 MSI imagery and existing monitoring station observations, to retrieve the COD concentration for each virtual sensor. Secondly, based on the virtual sensor network and monitoring stations, and Taylor expansion, we obtain a spatial estimation of COD in PL by solving a weighted least squares problem with the integration of the explanatory variables of COD. The framework of this method is presented in Figure 3.

#### 2.2.1. Designing a Virtual Sensor Network

A much denser sampling site is necessary to obtain reliable COD estimates. In this study, we first design an optimal virtual sensor network in each month. The aim of the network is to provide the number of sensors and the locations to obtain detailed COD concentration information and their variations. Optimization of a COD sampling network varies over time, including both the disposition of the sites and the number of them.

First, several candidate locations $\left\{S_{1},S_{2},\cdots ,S_{n}\right\}$ in areas without COD monitoring sensors are given, especially in the intersection of the rivers, complex terrain areas, and areas that are poorly monitored, using Creat Features Tools in ArcGIS 10.6. These candidate locations were evenly distributed over the water surface with a sample interval of 10 m, using the grid method in Sampling Tools in ArcGIS 10.6. We then applied Shannon’s entropy to design the optimal virtual sensor network, and the number of the sensors was determined by using semi-variogram function.

Entropy is used to measure the information of an event [39], and can be calculated as,

(2) $$E(S)=-\sum_{i}p_{i}\ln p_{i},$$

where $p_{i}$ is the probability of the event $k_{i}$. For the COD concentration, E(S) represents the average amount of COD. The overlapping information could be found in two COD sampling sites. The joint entropy of COD from two sensors $S_{1}$ and $S_{2}$ is,

(3) $$E(S_{1},S_{2})=-\sum_{i}\sum_{j}p_{{}_{ij}}\ln p_{ij}$$

To find the sensor with the smallest reduction in uncertainty, conditional entropy is used and is expressed as follows:

(4) $$E(S_{2}|S_{1})=E(S_{1}|S_{2})-E(S_{1})$$

According to the above equations, we then calculate the entropy of each candidate virtual sensor in PL and, first, find the site $S_{1}$ with the highest uncertainty,

(5) $$\max E(S_{i}),i=1,2,\cdots ,n$$

Determine the second important site, $S_{2}$, which has the largest difference from the first site $S_{1}$, from the remaining candidate sensors by using the following equation,

(6) $$\min\left\{E(S_{1})-E(S_{1}|S_{2})\right\}$$

Similarly, find the third most important virtual sensor such that,

(7) $$\min\left\{E(S_{1},S_{2})-E((S_{1},S_{2})|S_{3})\right\}$$

Repeat the process, the jth important sensor satisfies,

(8) $$\min\left\{E(S_{1},\cdots ,S_{j-1})-E((S_{1},\cdots ,S_{j-1})|S_{j})\right\}$$

The monthly average COD concentration of the sites $\left\{S_{1},S_{2},\cdots ,S_{n}\right\}$ can be calculated using the random forest (RF) method by combing different bands of Sentinel-2 imagery. Finally, by comparing the semi-variogram function of the existing monitoring stations with that of the virtual sensors, the number of virtual sensors was determined.

#### 2.2.2. Proposing a New Estimation Method for Generating Spatial Fields for COD

Let $g_{i}$ denote the i-th observation of COD concentration from the optimal COD network, u denote the final estimation of COD in PL. The following equation can be established,

(9) $$g_{i}=u(p_{i})+\varepsilon_{i},i=1,2,\cdots ,M$$

where, $\varepsilon_{i}$ is the error, M denotes the neighborhood sampling number. $p_{i}={\left(x_{i},y_{i}\right)}^{T}$ is the COD value from the near sampling point.

Suppose that $p={\left(x,y\right)}^{T}$ is the surrounding site of $p_{i}={\left(x_{i},y_{i}\right)}^{T}$, we can obtain the following equation based on Taylor expansion:

(10) $$\begin{array}{l} u(p_{i})\approx u(p)+{\left\{\nabla u(p)\right\}}^{T}(p_{i}-p)+\frac{1}{2}{\left(p_{i}-p\right)}^{T}\left\{Hu(p)\right\}(p_{i}-p)+\cdots \\ =u(p)+{\left\{\nabla u(p)\right\}}^{T}(p_{i}-p)+\frac{1}{2}vech^{T}\left\{Hu(p)\right\}vech\{(p_{i}-p){\left(p_{i}-p\right)}^{T}\}+\cdots \end{array}$$

where, ∇ and H represent the gradient operator and Hession operator [40], with dimension of 2×1, 2×2, respectively. vech denotes a matrix hemivectorization operator, converting a matrix to a vector in lexicographical order:

(11) $$vech\left(\left[\begin{array}{cc} a & b\\ b & d \end{array}\right]\right)={\left[\begin{array}{ccc} a & b & d \end{array}\right]}^{T},\;vech\left(\left[\begin{array}{ccc} a & b & c\\ b & e & f\\ c & f & i \end{array}\right]\right)={\left[\begin{array}{cccccc} a & b & c & e & f & i \end{array}\right]}^{T}$$

Let,

(12) $$\beta_{0}=u(p),\;\beta_{1}=\nabla u(p)={\left[\begin{array}{cc} \frac{\partial u(p)}{\partial x} & \frac{\partial u(p)}{\partial y} \end{array}\right]}^{T},\;\beta_{2}=\frac{1}{2}{\left[\begin{array}{ccc} \frac{\partial^{2}u(p)}{\partial x^{2}} & 2\frac{\partial^{2}u(p)}{\partial x\partial y} & \frac{\partial^{2}u(p)}{\partial y^{2}} \end{array}\right]}^{T},$$

Equation (10) can be rewritten as:

(13) $$u(p_{i})\approx \beta_{0}+{\left\{\beta_{1}\right\}}^{T}(p_{i}-p)+\frac{1}{2}{\left\{\beta_{2}\right\}}^{T}vech\left\{(p_{i}-p){\left(p_{i}-p\right)}^{T}\right\}+\cdots$$

where, $\beta_{0}$ is the COD estimate at the point $p={\left(x,y\right)}^{T}$.

Taking into account the spatial heterogeneity of COD, the above approach can be expressed as a weighted least squared problem by introducing a weighted matrix generated from the sampling points:

(14) $$\underset{u}{\min}\sum_{i=1}^{P}(g_{i}-u(p_{i}){)}^{2}\cdot K_{W}(p_{i}-p)$$

where, $K_{W}(p_{i}-p)=\frac{1}{\det(W)}K(W^{-1}(p_{i}-p))$, K is a two-dimensional local windowing kernel function and is mainly used to consider the weight between the points in the local window and the current sampling points. If the distance is far, the weight is small, otherwise, the weight is large. W determines the kernel’s support set, with the simplest case of W=σI, σ is a global smoothing parameter.

In this study, for each sampling point $p_{i}={\left(x_{i},y_{i}\right)}^{T}$, we design W as a symmetric positive definite matrix with controllable direction by combining the explanatory variables of COD, as follows:

(15) $$W_{i}=\sigma C_{i}^{-1/2}$$

where, $C_{i}={\left(X^{T}(F_{i}(x_{i},y_{i}))X\right)}^{-1}X^{T}F_{i}(x_{i},y_{i})$ is given by the dominant covariates of COD selected from turbidity, PH, water temperature, dissolved oxygen, precipitation, and wind in surrounding area of $g_{i}$. X is composed of the covariates identified using RF method. Suppose the local neighborhood of $p_{i}={\left(x_{i},y_{i}\right)}^{T}$ is $N(p_{i})$, M is the sampling point number in $N(p_{i})$, a derivative matrix with dimension of M×2 can be calculated as:

(16) Ji=⋅⋅⋅⋅∂u(pi)∂x∂u(pi)∂y⋅⋅⋅⋅, pj∈N(pi),j=1,2,⋯,M

Then, let $F_{i}$ be the covariance matrix of the gradients in the local neighborhood:

(17) $$F_{i}=J_{i}^{T}J_{i}=\left[\begin{array}{cc} \sum_{j=1}^{M}{\left(\frac{\partial u(P_{j})}{\partial x}\right)}^{2} & \sum_{j=1}^{M}\frac{\partial u(P_{j})}{\partial x}\frac{\partial u(P_{j})}{\partial y}\\ \sum_{j=1}^{M}\frac{\partial u(P_{j})}{\partial x}\frac{\partial u(P_{j})}{\partial y} & \sum_{j=1}^{M}{\left(\frac{\partial u(P_{j})}{\partial y}\right)}^{2} \end{array}\right].$$

Combining Equations (13) and (14), we can get:

(18) $$\underset{{\left\{\beta_{n}\right\}}_{n=0}^{N}}{\min}\sum_{i=1}^{M}(g_{i}-\beta_{0}-{\left\{\beta_{1}\right\}}^{T}(p_{i}-p)-\frac{1}{2}{\left\{\beta_{2}\right\}}^{T}vech\{(p_{i}-p){\left(p_{i}-p\right)}^{T}\}-\cdots {)}^{2}\cdot K_{W}(p-p_{i})$$

Let,

(19) $$g={\left[\begin{array}{cccc} g_{1} & g_{2} & \cdots & g_{M} \end{array}\right]}^{T}\;b={\left[\begin{array}{cccc} \beta_{0} & \beta_{1}^{T} & \cdots & \beta_{N}^{T} \end{array}\right]}^{T}\;K=diag\left[\begin{array}{cccc} K_{W}(p_{1}-p) & K_{W}(p_{2}-p) & \cdots & K_{W}(p_{M}-p) \end{array}\right]\;X=\left[\begin{array}{cccc} 1 & {\left(p_{1}-p\right)}^{T} & vech^{T}\{(p_{1}-p){\left(p_{1}-p\right)}^{T}\} & \cdots \\ 1 & {\left(p_{2}-p\right)}^{T} & vech^{T}\{(p_{2}-p){\left(p_{2}-p\right)}^{T}\} & \cdots \\ \vdots & \vdots & \vdots & \vdots \\ 1 & {\left(p_{M}-p\right)}^{T} & vech^{T}\{(p_{M}-p){\left(p_{M}-p\right)}^{T}\} & \cdots \end{array}\right]$$

where, N is the dimension, diag denotes a diagonal matrix.

Equation (18) can be finally transformed into:

(20) $$\bar{b}=\arg\underset{b}{\min}\{{\left\|g-Xb\right\|}_{K}^{2}={\left(g-Xb\right)}^{T}K(g-Xb)\}$$

The solution of Equation (20) can be written as:

(21) $$\bar{b}={\left(X^{T}KX\right)}^{-1}X^{T}Kg$$

Therefore, the COD value can be obtained from the weighted combination of adjacent sampling points:

(22) $$\beta_{0}=e_{1}^{T}\bar{b}=\sum_{i=1}^{M}w_{i}(K_{W},N,p_{i}-p)g_{i}$$

where, $\sum_{i=1}^{M}w_{i}(K_{W},N,p_{i}-p)=1$, $e_{1}^{T}=\left[\begin{array}{cccc} 1 & 0 & \cdots & 0 \end{array}\right]$, $w_{i}(K_{W},N,p_{i}-p)$ is the equivalent kernel of $g_{i}$ and can be calculated from the elements of $X^{T}KX$.

The proposed method using the Taylor expansion above was based on spatial correlation and spatial heterogeneity of the variables by considering the points and their surrounding observations together with the environmental influence factors, and was named TSCH.

### 2.3. Model Performance Assessment

We use the leave-one cross validation method to evaluate the performance of TSCH approach. By using this method, only one sample point is used to validate the method and the remaining samples are used to train the method. This process repeats until each sample from the dataset is used as a validation set. The performance of TSCH is quantified by averaging the commonly used error measurements calculated from the cross-validation procedures, including the coefficient of determination (R^2^), mean absolute error (MAE), and root mean square error (RMSE). These metrics are defined by the following Equations (23)–(25), respectively.

(23) $$R^{2}=1-\frac{\sum_{i=1}^{m}{\left(y_{{}_{i}}^{*}-y_{i}\right)}^{2}}{\sum_{i=1}^{m}{\left(y_{{}_{i}}^{*}-{\bar{y}}^{*}\right)}^{2}}$$

(24) $$\mathrm{MAE}=\frac{\sum_{i=1}^{m}\left|y_{i}-y_{i}^{*}\right|}{m}$$

(25) $$\mathrm{RMSE}=\sqrt{\frac{\sum_{i=1}^{m}{\left(y_{i}-y_{i}^{*}\right)}^{2}}{m}}$$

where, m is the data number. $y_{i}$ and $y_{i}{}^{*}$ are the estimate and observation at the *i*th point, respectively. $\bar{y}$ and ${\bar{y}}^{*}$ are the average of $y_{i}$ and $y_{i}^{*}$, and $\sigma_{y}$ and $\sigma_{y^{*}}$ are the standard deviation of $y_{i}$ and $y_{i}^{*}$, respectively.

## 3. Results

**Figure 4:**
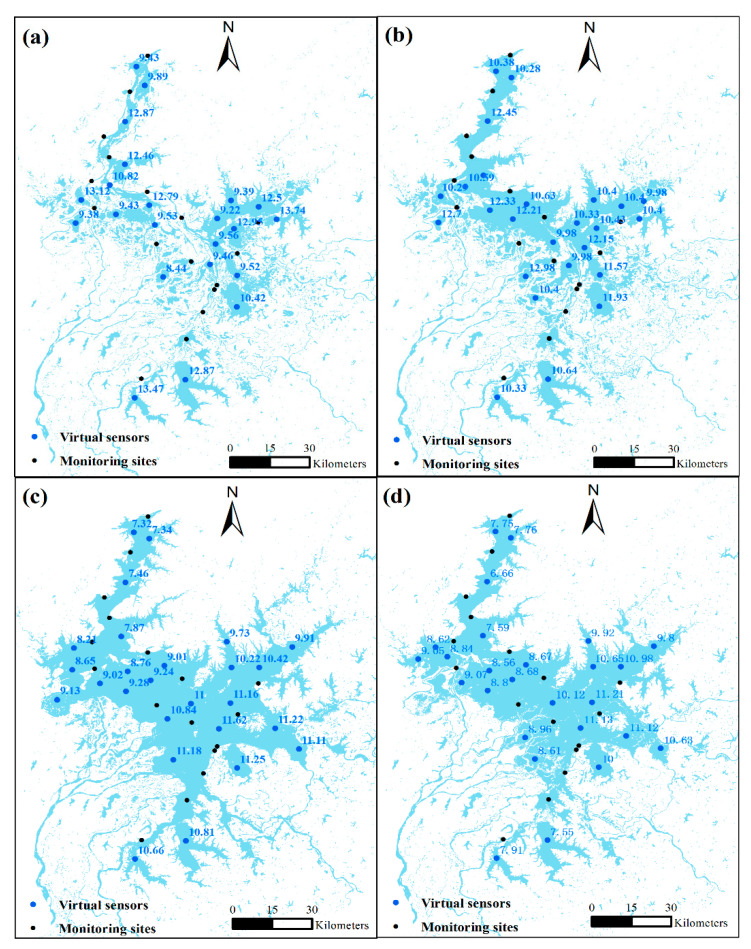
The distribution of virtual sensors (blue points) and the associated COD concentration values: (**a**) January; (**b**) April; (**c**) July; (**d**) October.

Based on the Shannon’s entropy and semi-variogram function, the optimal virtual sensor networks were established. Since the water areas fluctuated largely over the months, the network with the largest number of sensors was first established on July. The sensor network was then individually established and adjusted according to the water area in other months based on the sensor network in July and the corresponding monitoring station values in the month. We take four months from four seasons in 2021 as examples in this study. The distribution of the optimal sensor network is shown in Figure 4. To find the optimal Sentinel-2 image band compositions for COD retrieval, we compared 255 possible band combinations using RF method based on R^2^. The optimal band combinations for COD retrieval in January, April, July, and October (R^2^ > 0.75) were ‘Green + nir + red + red1’, ‘blue + green + red1 + red2’, ‘blue + green + nir + red2’, and ‘nir + red + red2 + swir1’, respectively. The COD concentration values for the established virtual sensors were obtained using these band compositions from Sentinel-2 imagery (Figure 4).

**Figure 5:**
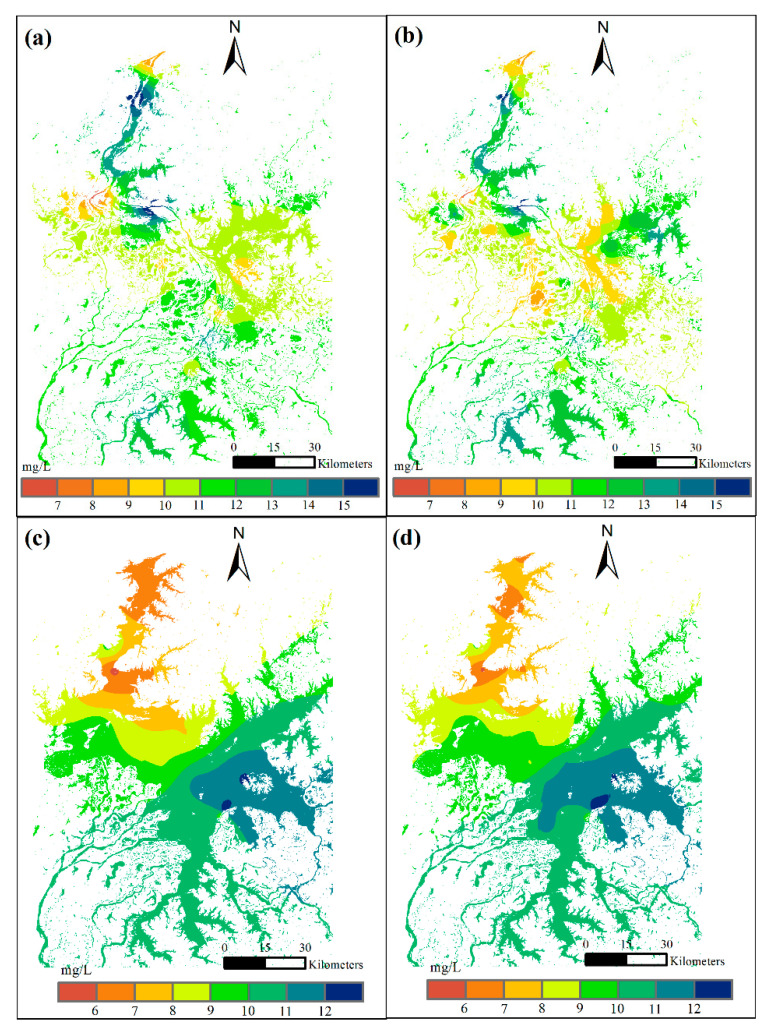
The spatial patterns of COD concentrations in two example months in dry and wet seasons: (**a**) January with monitoring sites only; (**b**) January with additional virtual sensors; (**c**) July with monitoring sites only; (**d**) July with additional virtual sensors.

The spatial distributions of COD concentrations in January and July were shown in Figure 5. Comparisons were made between estimates using only monitoring stations (Figure 5a,c) and using both monitoring sites and virtual sensors (Figure 5b,d). The results show that the additional virtual sensors generated similar spatial patterns of COD concentrations to the estimations using only monitoring sites. However, large local differences were observed in both two example months. In January, the mid-east, mid-west, and middle areas exhibited different spatial patterns before and after the use of virtual sensors (Figure 5a,b). The COD values were higher in mid-eastern and mid-western parts, while COD values were smaller in the middle PL after using virtual sensors. It should be noted that in the dry season, low precipitation and slow water flow in PL lead to relatively heterogeneous COD values, which were better reflected by using virtual sensors together with monitoring sites (Figure 5b). In July, local differences in COD values were observed in the north and south-central PL. Larger areas with larger values were observed in the south-central part and smaller values were observed in the northern part after considering the virtual sensors (Figure 5d). Overall, COD concentrations ranged from 5 to 13 mg/L, decreasing from south to north in July, with the highest values occurring in the south-central part.

**Figure 6:**
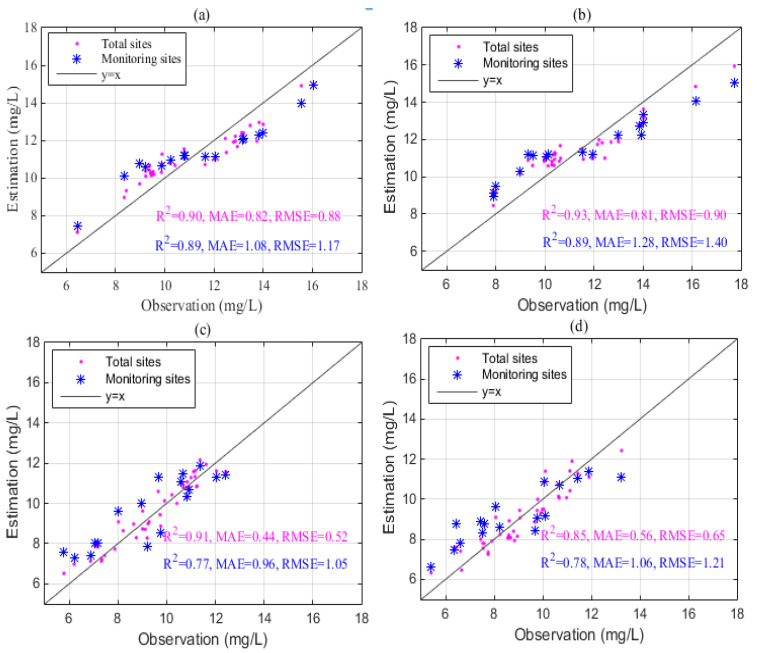
The comparison between estimations and observations in (**a**) January, (**b**) April, (**c**) July, and (**d**) October.

The model performance was quantified using R^2^, MAE and RMSE. Figure 6 presents the scatterplot of estimations and observations over the four example months. The results showed that TSCH had good COD estimation performance. COD estimates from both virtual and monitoring sensors agree well with observations based on R^2^, MAE, and RMSE (Figure 6). In January, the proposed method integrated with the virtual sensors generated better COD estimates with R^2^ of 0.90, MAE of 0.82, and RMSE of 0.88, improved by 24% in terms of MAE by comparing with the case of monitoring sites. The proposed method tended to underestimate high COD values and overestimate low COD values in the dry season (Figure 6a,b). In July and October, the method showed better performance than in January and April. In addition, by integrating virtual sensors, the estimations of COD exhibited high accuracy with R^2^ of 0.91, MAE of 0.44 mg/L, and RMSE of 0.52 mg/L, improved by 20%, 54%, and 50%, respectively in July. It was noted that the proposed method exhibited relatively better performance in wet months compared to dry months, which may be due to relatively heterogeneous spatial distributions of COD and high COD concentrations in the dry season.

**Figure 7:**
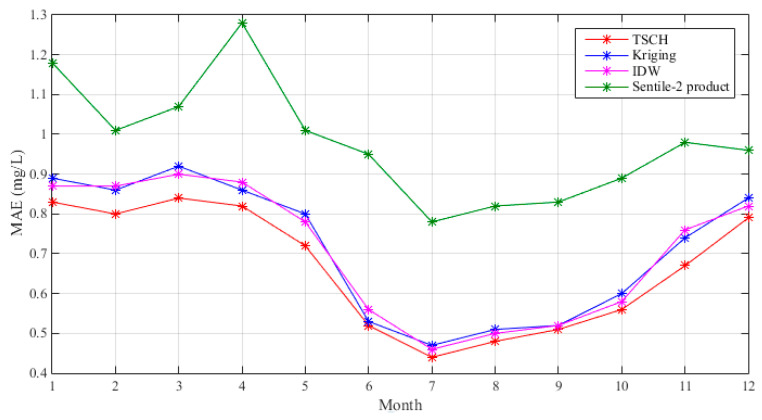
Comparison of different methods over 12 months.

The proposed method was compared with Kriging and inverse distance weighted (IDW) method and Sentinel-2 products over 12 months (Figure 7). Methods tended to produce better results in wet months than dry months. Based on MAEs, Sentinel-2 products exhibited the worst performance, while the proposed approach showed the best performance over the months. The poor performance of Sentinel-2 products may be due to weather conditions in different months and also the retrieval algorithms. Kriging performed better in some months than IDW, while IDW performed better in other months, and both performed worse than the proposed method. By considering the virtual sensors and environmental variables, the proposed method ensured the accuracy of the final COD concentration fields.

## 4. Discussion

Accurate estimating the spatial patterns of water quality parameters is critically important for water resource management and policy making. Although several remote sensors are available for generating water quality parameters at a large scale, estimating spatial patterns of these parameters is still a great challenge due to retrieval algorithms, sensors, and weather conditions. Numerical models can provide continuous spatial fields of water quality parameters, but have some uncertainties arising from the expression of the complex process and the parameterization. Traditional water quality monitoring provides relatively high accurate values of water quality parameters, but they just offer limited point values. Interpolators were the most common method of obtaining spatial distribution of water quality parameters. However, the accuracy of interpolation fields depends largely on the observation network.

The purpose of this study is to develop a novel approach for obtaining high accuracy spatial patterns of water quality parameters. Given the limited related work in PL, the developed approach was applied to generate COD fields in this area, and the results were critically important for monitoring the status of organic pollutant discharge in PL, and, thus, important for Poyang Lake wetland ecosystem and water resource management. We first established an optimal sensor network using entropy and semi-variogram function and employed and identified the optimal Sentinel-2 imagery band combinations to obtain COD values for each virtual sensor over different months. Considering Tobler’s first law of geography [41], a spatial estimation method was developed by using Taylor expansion among the optimal station network in each month. The method was thus transformed into a weighted least squares problem by considering the law of spatial heterogeneity with the integration of the explanatory variables of COD [42], selected from turbidity, PH, water temperature, dissolved oxygen, precipitation, and wind.

We evaluate the performance of the TSCH method by using leave-one cross-validation method. Results in Figure 6 and Figure 7 showed that TSCH can generate good COD concentration fields in PL in different months, and the method performed better in wet months when compared to dry months. The good performance of TSCH was mainly due to sufficient site values and integration with the explanatory variables of COD. Results in Figure 6 show that the additional virtual sensors provide better COD estimations, with MAE improved by 20% to 60% when compared to the method with monitoring sites alone. In addition, as shown in Figure 7, TSCH yields a significant improvement over satellite-based products, which may be due to the combination of monitoring values and the covariates of COD. The Sentinel-2 products exhibited high uncertainty and varied performance over the months, probably due to different weather conditions in PL. Results also show that the TSCH method performed better than other classical interpolators, with an average MAE improvement of 8% over the twelve months. These results also indicated that, although the satellite products were not satisfactory, the virtual sensor values, together with observations and explanatory variables, could together produce good results by using the proposed approach.

The proposed approach can be performed for other estimates of water quality parameters and can be applied to other domains, particularly to poorly monitored areas. In addition, the method of establishing the virtual sensor network can be used to design sampling point networks in other related studies. Despite the improved estimates, there are still some uncertainties in the final results. The accuracy calculated based on the leave-one cross-validation method varies with the location and the number of stations. TSCH can be found to be more suitable for COD estimation in a high-density station network. Although interpolation methods, such as Kriging and IDW, may also have good performance in data-intensive regions [43], the integration of explanatory variables in TSCH makes it better in estimating COD fields. The performance of TSCH was affected not only by the virtual sensor network, including virtual sensor locations, satellite image quality, and the number of monitoring stations used to train the RF method, but also by explanatory variables. The virtual sensor network should vary with spatial and time scales in applications. Given the higher heterogenous distributions of COD in the dry season, TSCH can be further improved by considering more explanatory variables.

## 5. Conclusions

In this study, a novel spatial estimation method, TSCH, was proposed for obtaining highly accurate water quality parameters. Entropy and semi-variogram were first employed to design an optimal virtual sensor network, and values of the virtual sensors were obtained using Sentinel-2 products. A Taylor expansion-based method was then developed using the optimal station network, with the integrating of spatial correlation and spatial heterogeneity of the variables. The TSCH method was used to obtain the COD fields in PL and the strict cross-validation results show that the COD estimates derived from the proposed approach agree well with the observations based on R2, MAE, and RMSE. TSCH performed better than other classic interpolators, with MAE improved by 8%, and virtual sensors played an important role in COD estimation, with MAE improved by 20% to 60% when compared to the method with monitoring sites alone. The proposed method provides a promising way to obtain high-quality water quality parameters and can be applied to other environmental variables.

## Data Availability

Data and additional information can be provided by directly contacting the author.
